# Supplementary material for: Genome-Wide Analysis of the LRR-RLP Gene Family in a Wild Banana (Musa acuminata ssp. malaccensis) Uncovers Multiple Fusarium Wilt Resistance Gene Candidates
Source: Genes (Basel). 2022 Apr 2;13(4):638. doi: 10.3390/genes13040638 (PMC9025879; doi:10.3390/genes13040638)
Supplement: Supplementary file 1 [file genes-13-00638-s001.zip › genes-1647111-supplementary.pdf]

Table S1. LRR-RLPs with known functions in plant immunity or development used in this study for HMM profile construction and phylogenetic analyses.

Gene	Genbank accession number	Specie	RLP Structure*	Protein length (aa)	Subcellular location	Function	Reference
Cf-9	AAA65235	*Solanum pimpinellifolium*	SP-LRR-TM	863	Cell membrane	Plant immunity	[64]
Cf-2	AAC15779	*Solanum pimpinellifolium*	SP-LRR-TM	1112	Cell membrane	Plant immunity	[65]
Cf-4	CAA05268	*Solanum habrochaites*	SP-LRR-TM	806	Cell membrane	Plant immunity	[66]
Cf-5	AAC78591	*Solanum lycopersicum*	SP-LRR-TM	968	Cell membrane	Plant immunity	[67]
CLV2	AAF02654	*Arabidopsis thaliana*	SP-LRR-TM	720	Cell membrane	Development	[68]
Fea2	AAL17871	*Zea mays*	SP-LRR-TM	613	Cell membrane	Development	[69]
Hcr9-4E	CAA05269	*Solanum habrochaites*	SP-LRR-TM	855	Cell membrane	Plant immunity	[70]
EIX2	AAR28378	*Solanum lycopersicum*	SP-LRR-TM	1021	Cell membrane	Plant immunity	[71]
HcrVf2	CAC40826	*Malus floribunda*	SP-LRR-TM	980	Cell membrane	Plant immunity	[72]
Ve1	ACR33106	*Solanum lycopersicum*	SP-LRR-TM	1053	Cell membrane	Plant immunity	[73]
ReMAX	NP_001322741	*Arabidopsis thaliana*	SP-LRR-TM	1077	Cell membrane	Plant immunity	[74]
RFO2	NP_173168	*Arabidopsis thaliana*	LRR-TM	756	Cell membrane	Plant immunity	[49]
RLP30	NP_187187	*Arabidopsis thaliana*	SP-LRR-TM	786	Cell membrane	Plant immunity	[75]
RBPG1	NP_189138	*Arabidopsis thaliana*	SP-LRR-TM	890	Cell membrane	Plant immunity	[76]
ELR	QED40966	*Solanum microdontum*	SP-LRR-TM	1093	Cell membrane	Plant immunity	[77]
RLM2	AJG42078	*Brassica napus*	SP-LRR-TM	925	Cell membrane	Plant immunity	[78]
CuRe1	XP_025888168	*Solanum lycopersicum*	SP-LRR-TM	1121	Cell membrane	Plant immunity	[79]
I-7	AKR80573	*Solanum pennellii*	SP-LRR-TM	966	Cell membrane	Plant immunity	[22]
I	Solyc11g011180	*Solanum pimpinellifolium*	SP-LRR-TM	994	Cell membrane	Plant immunity	[23]
RLP23	NP_180827	*Arabidopsis thaliana*	SP-LRR-TM	890	Cell membrane	Plant immunity	[80]

* SP, signal peptide; LRR, leucine rich repeat domain; TM, transmembrane domain. The signal peptide and protein domains were identified with the SMART and InterProScan programs. The subcellular localization was predicted using the DeepLoc program.

64. Jones, D.A.; Thomas, C.M.; Hammond-Kosack, K.E.; Balint-Kurti, P.J.; Jones, J.D.G. Isolation of the tomato Cf-9 gene for resistance to Cladosporium fulvum by Transposon Tagging. Science 1994, 266, 789–793. https://doi.org/10.1126/science.7973631.

65. Dixon, M.S.; Jones, D.A.; Keddie, J.S.; Thomas, C.M.; Harrison, K.; Jones, J.D.G. The tomato Cf-2 disease resistance locus comprises two functional genes encoding leucine-rich repeat proteins. Cell 1996, 84, 451–459. https://doi.org/10.1016/S0092-8674(00)81290-8.

66. Thomas, C.M.; Jones, D.A.; Parniske, M.; Harrison, K.; Balint-Kurti, P.; Hatzixanthis, K.; Jones, J.D. Characterization of the tomato Cf-4 Gene for resistance to Cladosporium Fulvum identifies sequences that determine recognitional specificity in Cf-4 and Cf-9. Plant Cell 1997, 9, 2209–2224. https://doi.org/10.1105/tpc.9.12.2209.

67. Dixon, M.S.; Hatzixanthis, K.; Jones, D.A.; Harrison, K.; Jones, J.D.G. The tomato Cf-5 disease resistance gene and six homologs show pronounced allelic variation in leucine-rich repeat copy number. Plant Cell 1998, 10, 1915–1925. https://doi.org/10.1105/tpc.10.11.1915.

68. Jeong, S.; Trotochaud, A.E.; Clark, S.E. The Arabidopsis CLAVATA2 gene encodes a receptor-like protein required for the stability of the CLAVATA1 receptor-like kinase. Plant Cell 1999, 11, 1925–1933. https://doi.org/10.1105/tpc.11.10.1925.

69. Taguchi-Shiobara, F.; Yuan, Z.; Hake, S.; Jackson, D. The fasciated ear2 gene encodes a leucine-rich repeat receptor-like protein that regulates shoot meristem proliferation in maize. Genes Dev. 2001, 15, 2755–2766. https://doi.org/10.1101/gad.208501.

70. Westerink, N.; Brandwagt, B.F.; De Wit, P.J.G.M.; Joosten, M.H.A.J. Cladosporium fulvum circumvents the second functional resistance gene homologue at the Cf-4 Locus (Hcr9-4E) by secretion of a stable Avr4E isoform. Mol. Microbiol. 2004, 54, 533–545. https://doi.org/10.1111/j.1365-2958.2004.04288.x.

71. Ron, M.; Avni, A. The receptor for the fungal elicitor ethylene-inducing xylanase is a member of a resistance-like gene fam-ily in tomato. Plant Cell 2004, 16, 1604–1615. https://doi.org/10.1105/tpc.022475.

72. Belfanti, E.; Silfverberg-Dilworth, E.; Tartarini, S.; Patocchi, A.; Barbieri, M.; Zhu, J.; Vinatzer, B.A.; Gianfranceschi, L.; Gessler, C.; Sansavini, S. The HcrVf2 gene from a wild apple confers scab resistance to a transgenic cultivated variety. Proc. Natl. Acad. Sci. USA 2004, 101, 886–890. https://doi.org/10.1073/pnas.0304808101.

73. Fradin, E.F.; Zhang, Z.; Ayala, J.C.J.; Castroverde, C.D.M.; Nazar, R.N.; Robb, J.; Liu, C.M.; Thomma, B.P.H.J. Genetic dissection of Verticillium wilt resistance mediated by tomato Ve1. Plant Physiol. 2009, 150, 320–332. https://doi.org/10.1104/pp.109.136762.

74. Jehle, A.K.; Lipschis, M.; Albert, M.; Fallahzadeh-Mamaghani, V.; Fürst, U.; Mueller, K.; Felix, G. The Receptor-like protein ReMAX of Arabidopsis detects the microbe-associated molecular pattern eMax from Xanthomonas. Plant Cell 2013, 25, 2330–2340. https://doi.org/10.1105/tpc.113.110833.

75. Zhang, W.; Fraiture, M.; Kolb, D.; Löffelhardt, B.; Desaki, Y.; Boutrot, F.F.G.; Tör, M.; Zipfel, C.; Gust, A.A.; Brunner, F. Arabidopsis RECEPTOR-LIKE PROTEIN30 and receptor-like kinase suppressor of BIR1-1/EVERSHED mediate innate immunity to necrotrophic fungi. Plant Cell 2013, 25, 4227–4241. https://doi.org/10.1105/tpc.113.117010.

76. Zhang, L.; Kars, I.; Essenstam, B.; Liebrand, T.W.H.; Wagemakers, L.; Elberse, J.; Tagkalaki, P.; Tjoitang, D.; van den Ackerveken, G.; van Kan, J.A.L. Fungal endopolygalacturonases are recognized as microbe-associated molecular patterns by the Arabidopsis receptor-like protein responsiveness to botrytis polygalacturonases1. Plant Physiol. 2014, 164, 352–364. https://doi.org/10.1104/pp.113.230698.

77. Du, J.; Verzaux, E.; Chaparro-Garcia, A.; Bijsterbosch, G.; Keizer, L.C.P.; Zhou, J.; Liebrand, T.W.H.; Xie, C.; Govers, F.; Robatzek, S.; et al. Elicitin recognition confers enhanced resistance to Phytophthora infestans in potato. Nat. Plants 2015, 1, 15034. https://doi.org/10.1038/nplants.2015.34.

78. Larkan, N.J.; Ma, L.; Borhan, M.H. The Brassica napus receptor-like protein RLM2 is encoded by a second allele of the LepR3/Rlm2 blackleg resistance locus. Plant Biotechnol. J. 2015, 13, 983–992. https://doi.org/10.1111/pbi.12341.

79. Hegenauer, V.; Fürst, U.; Kaiser, B.; Smoker, M.; Zipfel, C.; Felix, G.; Stahl, M.; Albert, M. Detection of the plant parasite Cuscuta reflexa by a tomato cell surface receptor. Science 2016, 353, 478–481.

80. Ono, E.; Mise, K.; Takano, Y. RLP23 is required for Arabidopsis immunity against the grey mould pathogen Botrytis Cinerea. Sci. Rep. 2020, 10, 13798. https://doi.org/10.1038/s41598-020-70485-1.

Table S2. Primers used for the RT-qPCR assays.

Primer name	Primer orientation	Primer sequence
MaLRR-RLP58 F	Forward	5′-ATGGCAGCATTCCGGAGGTG-3′
MaLRR-RLP58 R	Reverse	5′-TCAGTGATGCTAACGCTTGAG-3′
MaLRR-RLP74 F	Forward	5′-TTGCCTACGATGCTACCGTC-3′
MaLRR-RLP74 R	Reverse	5′-AAGGTCAAGGTATTCCAGACC-3′
MaLRR-RLP78 F	Forward	5′-TTGGTGCATCGGCGTACTG-3′
MaLRR-RLP78 R	Reverse	5′-TCTCCTGATCGATATCACTGC-3′

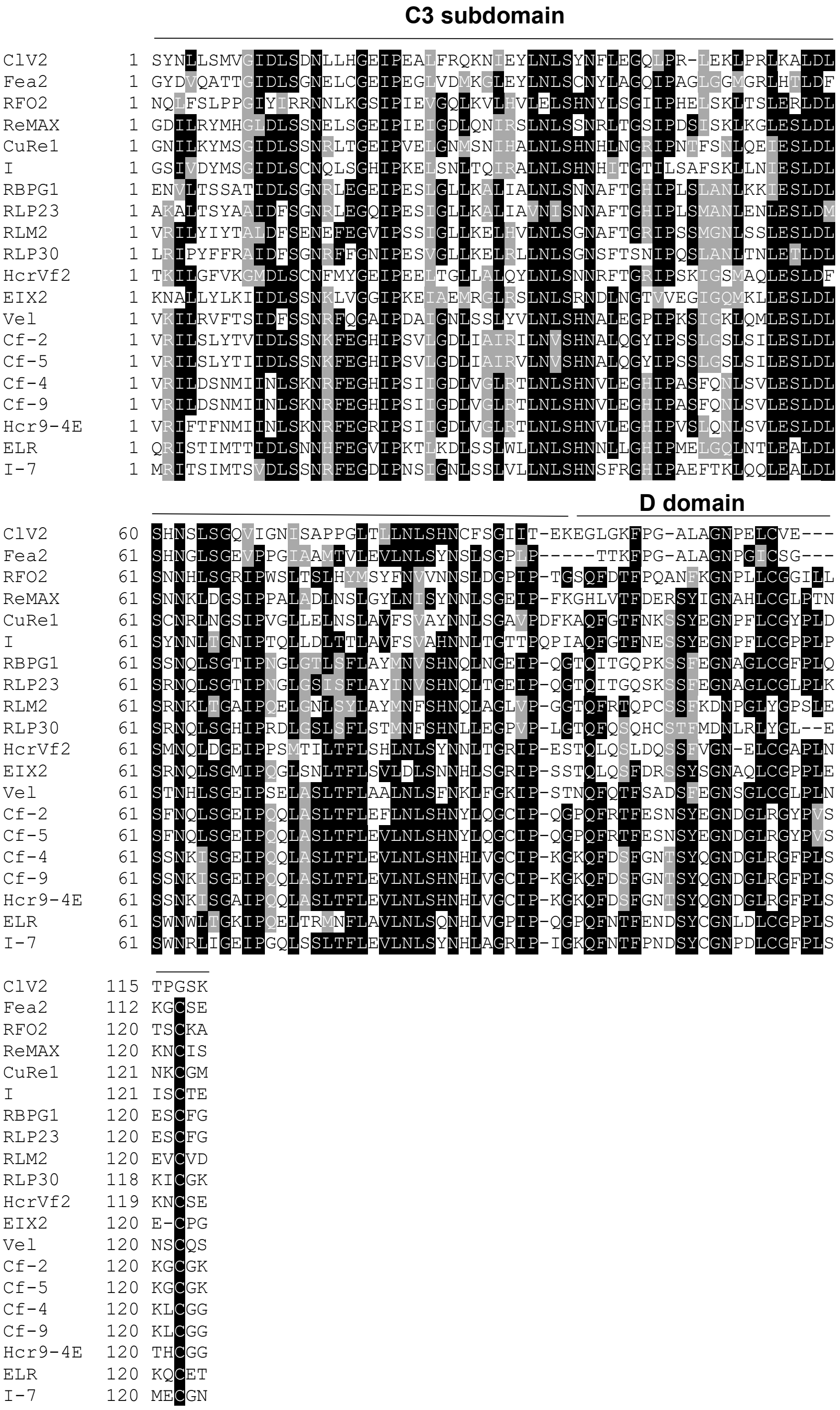

Figure S1. ClustalO alignment of the C3-D region of 20 LRR-RLPs with known functions. The C3 subdomain and D domain used by Kang and Yeom [27] are underlined. This region was used for the HMM profile construction. Identical amino acids are shaded in black, and conserved substitutions are shaded in gray.

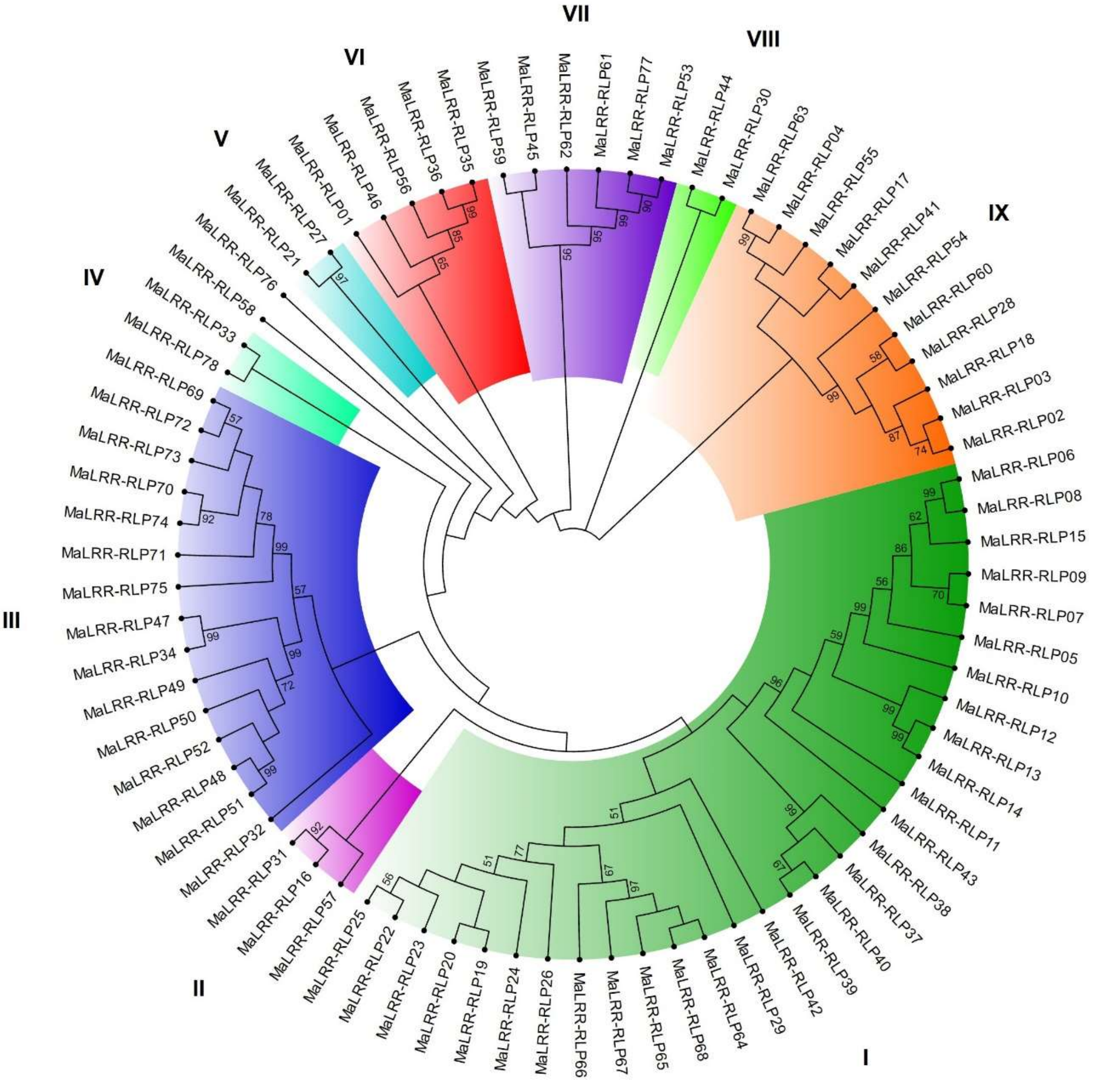

Figure S2. Maximum-likelihood phylogenetic tree of the banana LRR-RLP family. The roman numerals in bold indicate major clades of the phylogenetic tree. Numbers on the branches indicate the percentage of 100 bootstrap replications supporting the particular nodes, and only those ≥50% are shown. The C3-F region of LRR-RLPs was used for phylogenetic tree construction.

```
I               MT-------NSKIVYWWLLILFMANGWLCCYCCLNEEKTALLQLKANINYSTH---------DDYLSSWEANETSDCCQW 64
I-7             MEYQQL---LIAFCFYSLFVQQSQFAYAGKHLCPRDQAFYLLQFKQGLTVDPNAYDCENKARSKTLSW---NVTGDCCEW 74
MaLRR-RLP74     MGFPLRFLSSLSLCLLALLLHRATVT----SGCFSMEREALLDFKAGIHDT-----------YNRLSS---WVGQDCCAW 62
MaLRR-RLP75     MGFPLRFLSSLLLCLLALLLQRATVT----SGCFSMEREALLDFKAGIHDT-----------QNRLSS---WVGQDCCAW 62

I               EGIVCSNSTRRVVELSVIAKQISQEEQLGNFTTRDDMLRNWLFNASLFIPFKNLKALNIPGHSLAGWVKNEGFEKLRPLR 144
I-7             DGVTCNGLTGHVIGLDLSCSFLI---------------GTINAN-----------------------------SSLTKLS 110
MaLRR-RLP74     EGVICGATTGHVVMLDLRNTNIYDWKL-----------RGERMN-----------------------------SSLLALS 102
MaLRR-RLP75     AGVICRATTGHVVKLDLRNTNTLDGAL-----------RGERMN-----------------------------SSLLALS 102

I               KLERLDLSGNQFNRS-IFQSLSQLSSLKSLNLSSNNFAPRSEM--------------------WFTDNKIGSGSER---- 199
I-7             HLQRLNLASNEFNDFPLGNSTSELSSLTHLNLSDSGIFNERKMIPPGLCKLSKLISLDLSGSYIQ--------VGRTTFT 182
MaLRR-RLP74     HLERLDLSFNDFSGIRIPEFIGSFKKLRYLNLSSTNFM---GGIPARLGNLSSLHVLDLSDALHFT-----SHVDNLHWL 174
MaLRR-RLP75     HLRHLDLSFNDFSGIRIPEFIGSFKKLRYLNLSSTYFM---GGIPARLGNLSSLYVLDLSDALYVDDYGNGYPVDNLDWL 179

I               --------------------------------------------------LSGLDKLEILDLSYNDLEDKKFLSALELNT 229
I-7             SLLHNLTNLEVLLFDYVYASFELPKKFPSSLRKLSLQGTNVFGN-ITDSQLFHLPNLQVLRLGDNPSLTGT-LPNFHWNF 260
MaLRR-RLP74     SHLTSL-------------------------KHLDLSGLNLTDVPDWFSSVNMLPSLQVLSMSSVGLDTIP-ASVVHINF 228
MaLRR-RLP75      SHLTSL-------------------------KHLDLSELNLTVVPDWFSSVNMLPSLQVLNMSYGGLNSIP-VSVVHVNF 233

I               SRPTLKKLNIRYSRFQSFIPNEELGALRNIEYLLLDGITLDKNFLRSSGVMSSLKVLSVAQCS-LNGTLPRQGLCDLKYL 308
I-7             -SKSVLE-------------------------LDFSYTGIFGKVPDSIGILQSLWRLNLFNCH-LSGSIPE-SFGNLTTI 312
MaLRR-RLP74     -TSSLTVLDLSYNHFDSTLPKW-LGNITSLTHLDLFYSGFYGVIPDAIGDLGSLTFLDLGDNQ-LEGTVPR-SMVDLRRL 304
MaLRR-RLP75     -TSSLTVLDLSFNSFSSTLPKW-LGNVSGLTSLGLSSCEFLGTIPDAIGDLSSLTFLNLGNNFLLEGIVPR-SMANLHRL 310

I               EELSLSRNSFTGKLPACLRNLTFLRVIDLTQNQFTGNIA------------------------LSPLSSLLSLEYLLITN 364
I-7             RELILSGNNFTGNILSTISKLNKLVNLHLSSNHFRGSIPESIGNLTAIRELILSDNSFAGYVPSS-IGKLNKLDSLSLSS 391
MaLRR-RLP74     KELHMQGNRLTGNLSDLLEQMTNLIILDLQSNLFNGSMPSSVGKFSNLTELNLAGNSVGGVLSEVHFENLTRLRLLDLSG 384
MaLRR-RLP75     KELDLSVNQLSGTLTGWLEQLTNLIVLNLSYNLFNGSVPASVGKFSNLTGLYLGGNSLGGVISEAHFESLTKLRVLDLFD 390

I               NNFEVPISFESFANHSKLKFVFADYNS-VIVQTTSKIWIPKFQLEALSLFNSSQMPNFLHSQHHLRLIRLSKCNIGGDFP 443
I-7             NNFEGSIPD-----------IFANFSELYSLDFHSNNFVGPFPYSIATL-------------THLDGLELQNNSLTGPLP 447
MaLRR-RLP74     NPITISIG---------------------------QSWVPPFQLRYVDL---------------------TKCQLGPQFP 416
MaLRR-RLP75     NSITVSIG---------------------------QSWVPPFQLRVIYL---------------------SNCHLGPQFP 422

I               NWLLENNPKLGEVYLDGNAFTGSLQLPFLPNLKAFDISNNKIRGQLPPNIGSIFPN-LVISTMSSNMLEGLLPSSFADMQ 522
I-7             SNISGFQ-----------------------NLFNLDLSFNHLTGATPPWLFQLPSL--MSLSVQANKFTGELPNELNRST 502
MaLRR-RLP74     EWLQFQT-----------------------QIEKLYLADCKIAGTMPAWLWNISSSTITALDLSNNQIGGKLPSSLKFTK 473
MaLRR-RLP75     EWLRFQT-----------------------QTQELTMHDCKIAATIPNWFWNFSSSTVTLVDLSDNRIGGRLPSSLSFAR 479

I               SLECLDLSYNKLKGELPTRLARKG---------------------SKLYLLRLSNNMLKGEIFPASANINN-FNYLYLDG 580
I-7             SVPFIDISYNNLHGEIPYWMLSMVMNSLDLSHNFLTGFEKQVWHSEYLSYLNLENNFLQGPLHQSICDLIN-LEFLILAQ 581
MaLRR-RLP74     LV-ILYLDSNRFEGPLPTML-PSTLDTLFLSNNSFTGQ-LPIWPD--VQSVALSDNMLDGGLSSSICQWTGGLEYLDLSN 548
MaLRR-RLP75     LE-ELYLDSNIFEGPLPAML-PSALDTLYLSNNSFTGQ-LPVWPH--VSLALLSNNMLDGDISSSICRWTY-LRYLVLSG 553

I               NNFSGPIPQTLSTAPLRTLDLSYNNLSGNIPSWLGLEVLDISENNLVGVIPSCFSSFRDLKHVYLSKNKLQGEFN--MFS 658
I-7             NNFNGSIPDCLGNSNR------------------LISILDLRMNNFHGEIPTFLP--RGLEYLGLYGNQLRGQVPRSLVN 641
MaLRR-RLP74     NKLLGEIPYCLGKSLQ------------------NLYILNLGKNHFSGEIPHTIGFLSGLWHLQLKNNSFSGELPLSLKN 610
MaLRR-RLP75     NNLFGEIPYCLGESLQ------------------DLSILSLANNHFSGEIPNTIGFLTELKLLQLKNNSFSGELPLSLQN 615

I               NSGLRVLDLGDNNFSGSIPKWLGS--SGIITLLLKGNNLQGTIPTELC--HASYLRIMDLSHNNLSGPIPRCFGNIMQQE 734
I-7             RTSLVALDLGNNKLNDTFPIWLE-KLPNLQVLILKSNLFHGPIGDLESEFPFPELRIFDLSFNGFTGTLSSNLFKSFRGM 720
MaLRR-RLP74     CIGLRFLDLAQNNFVGSITPWIGDNLQQLVVLRLRSNMFSGVIPWQLA--RFEKLQILDLANNNFSGSIPHNIGN-LSTM 687
MaLRR-RLP75     CTNLQFLDLAQNNFVGSIPFWMGENLSQLAVLRLRSNMFSGDIPWQLA--QLGRLQILDLANNTFSGSIPHNFGN-FSTM 692

I               DIIELYPYSSSFGSSVFETFGGDTAIEVESSIMSSSTLLLLDNYVWVGAEFMTKHNTYSYEGSIVDYMSGIDLSCNQLSG 814
I-7             MDA----------------DEGKSG-------ISRARNRTRRDYLYHV-SLVIKGNEFD--MRITSIMTSVDLSSNRFEG 774
MaLRR-RLP74     RST-----------------------------------SQYNDFCYDELDVFTKGQDLYYLKCSIKLMKSMDLSNNSLTG 732
MaLRR-RLP75     AST-----------------------------------SDNGTFIYDLFDISTKGQDLFYIK-DLDLLKSLDLSNNRLIG 736

I               HIPKELSNLTQIRALNLSHNHITGTILSAFSKLLNIESLDLSYNNLTGNIPTQLLDLTTLAVFSVAHNNLTGTTPQPIAQ 894
I-7             DIPNSIGNLSSLVLLNLSHNSFRGHIPAEFTKLQQLEALDLSWNRLIGEIPGQLSSLTFLEVLNLSYNHLAGRIPIGK-Q 853
MaLRR-RLP74     EIPKGIGDLAGLKNLNLSRNYLQGKIPWEIGGMKSLESLDLSINDLSGSIPESLSVLYSLSYLNLSYNNLSGRIPTGR-Q 811
MaLRR-RLP75     DIPEEIGDLAGLKNFNLSRNHLKGKIPSQIGGMKLLESLDLSINDLSGSIPESLSALYFLSYLNLSYNNLSGRIPTGH-Q 815

I               FGTF-NESSYEGNPFLCGPPLPISCTEPKEIPKSPLEPECCEDDTGFLD-IELFYISFLVAFANV---VLATVVVLWVNP 969
I-7             FNTFPND-SYCGNPDLCGFPLSMECGNNNESPLERDDSDDDDDDSFFMSGFTWEAVAIGYGCGMIFGLLIGALMFLLEKP 932
MaLRR-RLP74     LQTLNDPSIYMGNADLCGPPTSKSCFDNKTTQIDIQEHEKE------ISDWLWFYISLVLGFVMGFWIFFGILF---LKD 882
MaLRR-RLP75     LQTLNDPSIYMGNANLCGPPTSKSCHNNKTAQNNIQEYEKE------ISEWLWFYISMVLGFVMGFWTFCGILF---LKD 886

I               YWRNVWFYYVEYYMYSCYYFFASK---M--------- 994
I-7             KW---YVKFAEDIAQQIAAKKRTRQKKIRQRRGVRMN 966
MaLRR-RLP74     AWRHAYFHFIDDVYDWVWVQ*---------------- 903
MaLRR-RLP75     AWRYAYFRMIDDMYDRIWVQWQLILRRLFER*----- 918
```

Figure S3. ClustalO alignment of I-7, I, MaLRR-RLP74 and MaLRR-RLP75. Identical amino acids are shaded in black and conservative substitutions are shaded in gray.
